# Multigene Family of Pore-Forming Toxins from Sea Anemone *Heteractis crispa*

**DOI:** 10.3390/md16060183

**Published:** 2018-05-24

**Authors:** Elena Leychenko, Marina Isaeva, Ekaterina Tkacheva, Elena Zelepuga, Aleksandra Kvetkina, Konstantin Guzev, Margarita Monastyrnaya, Emma Kozlovskaya

**Affiliations:** 1 G.B. Elyakov Pacific Institute of Bioorganic Chemistry, Far Eastern Branch, Russian Academy of Sciences, 159, Pr. 100 let Vladivostoku, Vladivostok 690022, Russia; issaeva@gmail.com (M.I.); estkacheva@gmail.com (E.T.); zel@piboc.dvo.ru (E.Z.); sashaledy@gmail.com (A.K.); k.guzev@gmail.com (K.G.); rita1950@mail.ru (M.M.); kozempa@mail.ru (E.K.); 2 School of Natural Sciences, Far Eastern Federal University, Sukhanova Street 8, Vladivostok 690091, Russia

**Keywords:** sea anemones, actinoporins, pore-forming toxins, multigene families

## Abstract

Sea anemones produce pore-forming toxins, actinoporins, which are interesting as tools for cytoplasmic membranes study, as well as being potential therapeutic agents for cancer therapy. This investigation is devoted to structural and functional study of the *Heteractis crispa* actinoporins diversity. Here, we described a multigene family consisting of 47 representatives expressed in the sea anemone tentacles as prepropeptide-coding transcripts. The phylogenetic analysis revealed that actinoporin clustering is consistent with the division of sea anemones into superfamilies and families. The transcriptomes of both *H. crispa* and *Heteractis magnifica* appear to contain a large repertoire of similar genes representing a rapid expansion of the actinoporin family due to gene duplication and sequence divergence. The presence of the most abundant specific group of actinoporins in *H. crispa* is the major difference between these species. The functional analysis of six recombinant actinoporins revealed that *H. crispa* actinoporin grouping was consistent with the different hemolytic activity of their representatives. According to molecular modeling data, we assume that the direction of the N-terminal dipole moment tightly reflects the actinoporins’ ability to possess hemolytic activity.

## 1. Introduction

Actinoporins are biologically active polypeptides (17–20 kDa) that are found in sea anemones [1,2,3,4,5]. Their structure includes compact β-fold, without disulfide bonds, formed by 12 β-sheets and two α-helices, one of which, functional and more extended, is located at the N-terminus, and the second one, short, is at the C-terminus [5]. According to systematic classification IUBMB, actinoporins belong to the α-pore-forming toxins (α-PFTs) of the equinatoxin family 1.С.38 [3]. Mature actinoporins consist of 165–179 amino acids and are comprised together with actinoporin-like proteins and fungal fruit-body lectin family into the superfamily of membrane binding proteins, called AF domains [6].

The actinoporin family is one of the four most common toxin protein families that are isolated from sea anemones together with sea anemone sodium channel inhibitory toxin family, type I subfamily, sea anemone type 3 (BDS) potassium channel toxin family, and venom Kunitz-type family, sea anemone type 2 potassium channel toxin subfamily [7]. The actinoporins have mainly been found in the Actinioidea, as well as Metridioidea and Actinostoloidea superfamilies [7,8]. It has been shown that the actinoporins are produced as isoforms. Some of them, equinatoxins EqtI–EqtV from *Actinia equina* [9,10,11], magnificalysins HMgI–HMgIII and HmT from *Heteractis magnifica* [12,13,14], sticholysins StnI and StnII from *Stichodactyla helianthus* [15], actinoporins RTX-A, RTX-S, RTX-G, and RTX-SII from *Heteractis crispa* [16,17,18], cytolysins Or-A and Or-G from *Oulactis orientalis* [19,20], cytolysins Pstx-20A and Pst-I from *Phyllodiscus semoni* [21,22], and actinoporins Avt-I and Avt-II from *Actineria villosa* [22,23] are isolated and functionally characterized. However, there are a lot of actinoporin sequences that are deduced on the base of their genes.

Therefore, thirty-four different amino acid sequences were deduced from fifty-two nucleotide sequences of *Heteractis magnifica* toxins [24], and more than thirty actinoporins of *H. crispa* [8,18,20,25,26], as well as some actinoporins from twenty-four other species of sea anemones [8] were revealed. It was shown that the isoforms had many amino acidic substitutions that were located in important regions for pore formation. The genetic structure of actinoporins consists of a pre-propeptide and a mature region, and, therefore, they can be synthetized in the Golgi apparatus as precursor forms, followed by proteolytic processing during secretion [27]. It was hypothesized that sea anemones could have suffered duplication, conversion, and mutation of genes that produced multigene families as an efficient response to evolutionary pressure, leading to successful strategies of predatory and defensive function [27].

Due to pore-forming activity, actinoporins represent an important model for studying protein-membrane interactions. Actinoporins EqtII [5,28], StnI, StnII [29,30,31], and FraC [32,33,34] are mostly studied, including the resolution of their monomeric soluble three-dimensional structures. Despite the high sequence homology, the hemolytic activity of these compounds and even the manner of the interaction with the membrane are different. The pore-forming mechanism has been studied in detail, but some stages of the process are still being discussed [33,34,35,36,37,38,39,40,41]. To clarify the role of amino acid residues during membrane binding, wild-type actinoporins, as well as recombinant and mutant ones that are produced in *Escherichia coli* have been used. [11,29,35,36,37,39,42,43,44,45,46,47,48,49,50,51,52,53]. In general, the process of pore formation by actinoporins involves its binding to a sphingomyelin of cytoplasmic membranes through the aromatic POC site, transition of a N-terminal α-helical region (1–25 aa) to the lipid-water interface, oligomerization of 3–4, 8, or 9 monomers within the membrane interface, and the insertion of the N-terminal region into membrane hydrophobic core resulted in the creation of the functionally active protein-lipid pore [4]. Actinoporin conformational transformation from the soluble state to the membrane-binding one is a fundamental α-PFT property that is directed at the disruption of biological targets [41,54]. Certain attention is now directed to the investigation of the actinoporin action on target organs and different cell cultures, as well as to the creation of actinoporin immunoconjugates with different ligands for selective killing of parasite and tumor cells [55,56,57]. StnII encapsulated into liposome have been recently reported to function as an adjuvant inducing a robust specific CTL response [58]. Moreover, earlier we demonstrated that RTX-A from *H. crispa* exhibited an antitumor effect and suppressed IGF-induced tumor transformation of JB6P + Cl41 mouse epithelial cells [59]. This effect was found to be due to the induction of p53-independent apoptosis and the inhibition of the activity of the oncogenic nuclear factors AP-1 and NF-κB.

In this respect, we continued studying the actinoporins from *H. crispa* and could identify and characterize a large family proposed here as the Hct-A and Hct-S multigene family of actinoporins that are expressed in the sea anemone tentacles as prepropeptide-coding transcripts. Their considerable diversity at the mRNA level suggests that these sequences are subject to diversifying selection. The hemolytic activity of six recombinant actinoporins was confirmed in vitro. The electrostatic properties of actinoporins were discussed and key amino acid residues were identified on the base of computer modeling data.

## 2. Results and Discussion

### 2.1. Precursor Primary Structure

**Figure 1:**
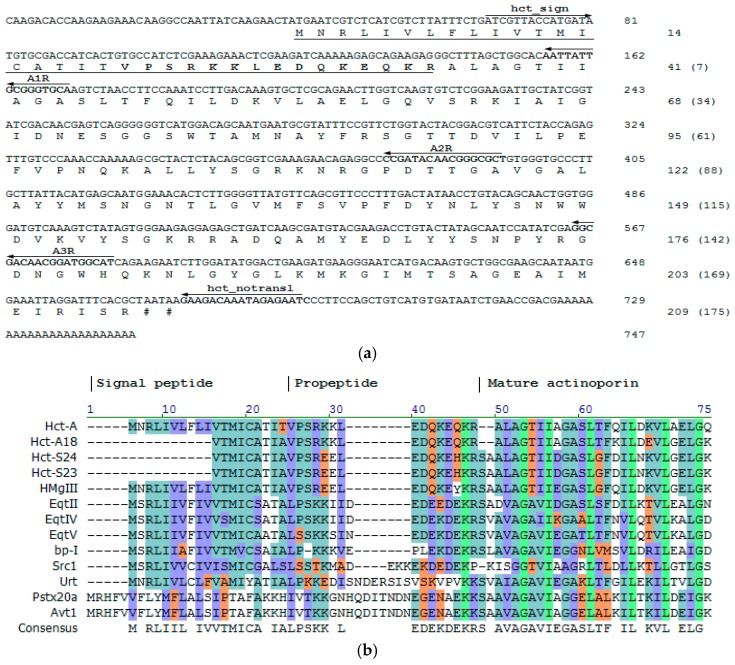
Structural organization of precursor proteins. (**a**) Nucleotide sequence of *hct-a* cDNA accompanied by deduced amino acid sequence. The signal peptide sequence is underlined, the propeptide sequence is bolded and underlined. The protein sequence is numbered starting from the presumed initiation methionine residue. The numbering of the mature polypeptide is in parentheses. The nucleotide sequences corresponding to primers A1R, A2R, A3R, hct_sign, and hct_notransl are bolded and are indicated by arrows. (**b**) Multiple sequence alignment for four actinoporin precursors from *H. crispa* and other sea anemones: HMgIII (Q9U6X1) from *H. magnifica*; EqtII (P61914), EqtIV (Q9Y1U9), and EqtV (Q93109) from *A. equina*; bp-1 (C5NSL2) from *A. asiatica*; Scr1 (Q86FQ0) from *S. rosea*; UcI (C9EIC7) from *U. crassicornis*; PsTX-20A (Q8IAE2) from *P. semoni*; Avt1 (Q5R231) from *A. villosa*; non-similar amino acids are shown on white background, weakly similar—on light-grown, conservative—on light-blue, block of similar—on purple, identical—on light-green colors. Gaps are represented by dashed lines. The boundaries of the signal peptide, prepropeptide, and N-terminal fragment of the mature protein are shown by vertical lines.

The full-length *hct-a* cDNA sequence was confirmed by the 3′- and 5′-RACE methods. In 3′-RACE, the PCR product was obtained using *H. crispa* cDNA as a template and degenerated and universal primers, as reported previously [18]. As a result of the Step-Out 5′-RACE technique [60] with the reverse A1R, A2R, and A3R and the universal RACE primers, the cDNA sequences (~200 bp) coding the signal peptide and containing 5′-untranslated region were obtained. More than 20 clones were sequenced and the sequences were not different. The full-length cDNA sequence, including 3′-untranslated region and a poly-A tail consisted of 747 bp, with an open reading frame of 627 bp and encoded an actinoporin precursor protein of 209 amino acid residues (Figure 1a). The translation start site (at the position 40–42 nt) contained highly conserved A residues of Kozak consensus at the position −3 and +4 [61]. The initiation start provided the translation of a typical signal peptide of 19 residues [62]. The propart sequence of 15 amino acid residues ending with Lys–Arg (a cleavage site) was located between the signal peptide and the mature region. The mature Hct-A consisted of 175 amino acids and was terminated by arginine.

As shown in Figure 1b, *H. crispa* actinoporin precursor has a classical structure consisting of prepropeptide and mature toxin region like precursors from different species of sea anemones. The actinoporin precursors from the sea anemones belonging to the Stichodactylidae family (actinoporins from *H. crispa* and *H. magnifica*) consist of an equal number of amino acids—19 aa in signal peptide and 15 aa in propeptide with 91% identity. Equinatoxin precursors (EqtII, EqtIV, and EqtV from *A. equina*, Actiniidae family) have 35 amino acids in the prepropeptide parts (19 aa in signal peptide and 16 aa in the propeptide, with identity ranging from 89 to 96%). Preproparts of the bandaporin, bp-1, from *Anthopleura asiatica* and the urticinatoxin, UcI, from *Urticina crassicornis* (Actiniidae family) consist of 34 and 42 aa, respectively. These sequences possess low identity (36–68% for bp-1 and 32–47% for UcI) both to the actinoporin precursors from the sea anemones of the same family and other actinoporin prepropeptides. Actinoporins from Aliciidae family, AvtI from *A. villosa,* and PsTX-20A from *P. semoni*, contain 47 amino acids in a prepropeptide part. These sequences show 100% identity to each other. However, their similarity with other actinoporin prepropeptides does not exceed 11%. In general, the actinoporin precursors have a conservative organization and they mainly consist of polar and charged amino acids. The similar precursor organization was also determined in sea anemone neurotoxins, cnidarian nematocyst collagens, and frog antimicrobial peptides, such as magainin and dermaseptin [63]. Propeptides are known to have various functions, including the assistance in folding, preventing biological activity, and precursor trafficking to different locations within a cell. The sequence—EDQKEQ/HKR—is a consensus for the most of actinoporin propeptides (Figure 1b). According to an assumption by Anderluh and coauthors, who have found a highly conservative sequence of nine residues (DEDEDIEKR) in the proparts of some toxin precursors and Cnidarian nematocyst collagens, the proparts play an important role in regulation of the secretory pathway [64].

### 2.2. Actinoporins Pertain to a Multigene Family

The full-length *hct-a* cDNA sequence was used to design gene-specific primers, which would allow for the detection of low-abundance transcripts, and hence minor actinoporin isoforms. To identify these sequences, two primers, hct_sign and hct_nontransl, were constructed on the base of a signal sequence and a 3′-untranslated region, respectively (Figure 1a). As the result, PCR fragments about 650 bp encoding the actinoporin precursors were cloned, and more than 100 clones were sequenced.

The forty-four different nucleotide sequences were determined, which could be translated into 43 distinct actinoporin precursors. The sequence identity was 91–99% at the nucleotide level, and 93–99% at the amino acid level. One sequence, Hct-A5, was encoded by two cDNAs differing in one synonymous substitution. All of the remaining sequences had only nonsynonymous substitutions. We previously reported that three isoforms, Hct-S5, Hct-S6, and Hct-S10, were also encoded by several genes [25]. The results indicate that *H. crispa* actinoporins are encoded by the multigene family containing of 47 representatives at least. Each of them was predicted to contain a signal peptide, a propart, and a mature protein.

The deduced mature actinoporins can been classified into two groups according to the different N-terminal residues: Hct-S (with serine residue) and Hct-A (with alanine residue). In total, we identified 25 Hct-S and 18 Hct-A isoforms (Figure S1). Some of them have been reported recently [25,26], whereas Hct-A7–Hct-A18 and Hct-S23–Hct-S28 were first found. The calculated values of the molecular masses of actinoporins were 19.12–19.52 kDa, and their isoelectric points were in the range of 9.10–9.74 that corresponded to those of native actinoporins [16,17,26].

**Figure 2:**
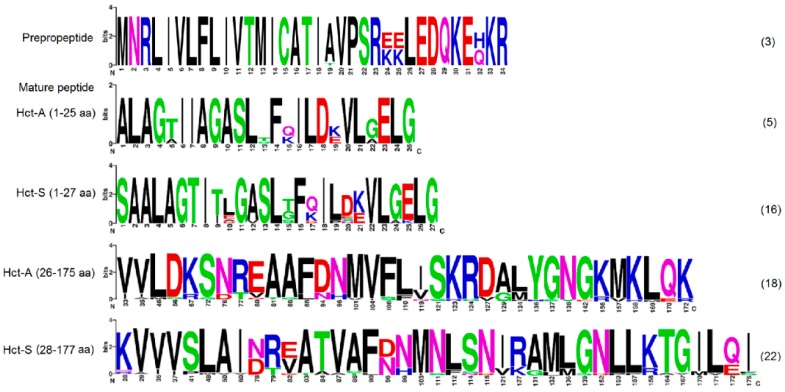
Sequence Logo visualization of the *H. crispa* actinoporin alignments. Mature sequence alignments (26–177 aa) are represented only in substitutive positions. Amounts of variable sequences are shown in parentheses to the right of the alignments.

Figure 2 shows the sequence Logo visualization of the *H. crispa* actinoporin alignments. The most sequence conservation is observed in the pre-propeptide accounted three polymorphic sites (A19T, 24EE(KK)25, and H32Q positions), which result in three sequence variants (Figure 1b). The rapid accumulation of amino acid substitutions occurs at the beginning of the mature protein (the first 27 residues) providing 5 Hct-A and 16 Hct-S variants. The remaining part of the mature protein contains greater numbers of substitutions, realizing additionally in 18 Hct-A and 25 Hct-S isoforms (Figure S1). This type of sequence conservation is also observed in many other toxins and immune-related peptides, such as antimicrobial peptides [65,66,67].

The sequence divergence of the mature actinoporins is also examined at the level of intra- and interspecific variation. In *Heteractis* genus, the both species *H. crispa* and *H. magnifica* [24] have much greater numbers of actinoporin genes, which strongly indicate multiple gene duplications and an existence of multiple isoforms. *Heteractis* actinoporins show comparable intraspecific values of genetic divergence, 0.069 ± 0.011 for *H. crispa* ones, and 0.072 ± 0.012 for *H. magnifica* ones. The inter-species divergence between actinoporins of *H. crispa* and *H. magnifica* is 0.094 ± 0.017. Interestingly, intraspecific distances between some *H. crispa* isoforms exceed interspecific divergence; for example, the genetic distance between RTX-A and Hct-S11 is 0.161. Comparative analysis of mature isoforms revealed that over a half (52.3%) of *H. crispa* sequences shared more than 95% identity to the *H. magnifica* ones, and only 2.2% of them were 100% identical. The 43.2% of sequences showed more than 90% identity. The data support close relationships between these species. We hypothesize that diversification of *Heteractis* actinoporins is driven by similar biotic interactions that has been recently shown that there is a clear correlation between venom composition and the interactions of sea anemones with prey and predator species [68]. Several actinoporin sequences have recently been deduced from *H. crispa* transcriptome by Macrander et al. [8]. Seventeen sequences have been revealed; six of them have corresponded to full-length mature actinoporins. However, we have not been able to find any identical or close sequences between their and our datasets. The sequence identity for all of these proteins has not exceeded 67.8%.

In the matter of multigene families encoding actinoporins, its existence has indeed been proved for many sea anemone species, and especially, the Stichodactylidae family ones. The first report was made for *S. helianthus*, up to 19 different cDNA sequences were detected [69]. Then, the multigene families of *H. magnifica* [24], *H. crispa* [25,26], and other sea anemones were found [8,70]. Indeed, actinoporin isoforms are produced in different amounts as it is shown for the copies of the actinoporin genes of *S. haddoni* [70]. However, the existence of actinoporin combinatorial libraries and an isoforms ratio in them remain unclear. A few different isoforms, for most sea anemones, are shown to be usually produced by the same species in large enough amounts for detection and purification [9,10,11,12,13,14,15,16,17,18,19,20,27]. The combinatorial library of actinoporins in *S. haddoni* venom has not also been found; the authors have identified only one actinoporin, although, according to 2DE data, actinoporins were found in many different spots [70]. Nevertheless, we have recently revealed the combinatorial library of *H. crispa* actinoporins by HPLC and MS, including 20 isoforms at least [26].

### 2.3. Phylogenetic and Sequences Analysis of Actinoporins

The evolutionary relationships of the actinoporins were inferred based on the mature protein sequences using the maximum likelihood (ML) method [71]. The best evolution model for the current dataset of sea anemone actinoporins was JTT + G + I. Evolutionary distances were calculated according to the JTT matrix-based model with a gamma distribution on the ungapped multiple alignment positions. The distances refer to the number of amino acid substitutions per site between two protein sequences (Table S1).

**Figure 3:**
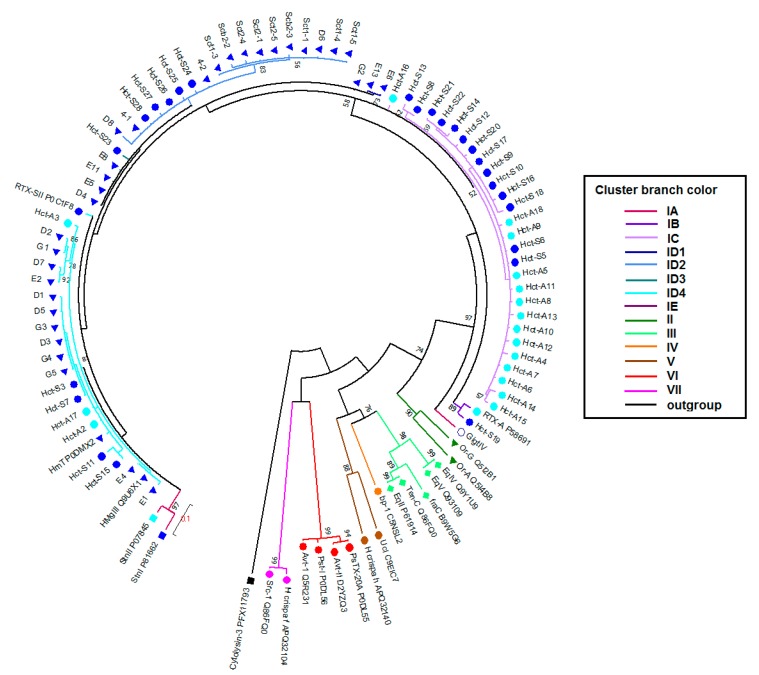
Phylogenetic analysis of actinoporins. The maximum likelihood (ML) phylogenetic tree was constructed using JTT model and pairwise distances with bootstrap support of 200 replications [71]. Nodes with confidence values greater than 50% are indicated. Clusters and groups are shown by colored branches. The *H. crispa* and *H. magnifica* actinoporins with N-end Ser residue are shown in dark blue circles and triangles, respectively, and with N-end Ala one in light blue circles.

According to the topology of the ML (under JTT model) tree, sea anemone actinoporins fall into seven clusters (I–VII) (Figure 3). Cluster I accounts the actinoporins of Stichodactylidae; the clusters II–V include the actinoporins of Actiniidae; and, the clusters VI and VII consist of the actinoporins of Aliciidae and Sagartiidae, respectively. The actinoporin-like polypeptide from coral *Stylophora pistillata*, cytolysin-3, is chosen as an outgroup [72]. The inter-cluster divergence values range from 0.290 ± 0.043 (between the clusters III and IV) until 0.567 ± 0.066 (between the clusters II and VI), whereas the intra-cluster divergence ones vary from 0.012 ± 0.008 (cluster VI) to 0.290 ± 0.044 (cluster V). Each cluster embraces actinoporin sequences (both orthologues and paralogues) of phylogenetically close species from the same family.

It is not surprising that *H. crispa* actinoporins f and h [8] do not group with any *H. crispa* actinoporin sequences due to their very low sequence identity (Figure S2). The fact that they fall in phylogenetically distant clusters V (Actiniidae) and VII (Sagartiidae) discourages us. The sequences share almost 100% identity with actinoporins of *Epiactis japonica* and *Sagartia elegans* [8]. The reasons for such kind of phenomenon might be misidentification and mixed sampling. Therefore, we did not use these sequences describing the phylogenetic relationships.

The cluster I consists of actinoporins of *Stichodactyla* and *Heteractis* genus, which could be split into five groups, IA–IE. The sequence identity between the cluster members ranges from 83 to 100% (Figure S2). The level of intra-cluster divergence is 0.080 ± 0.013 with a maximum value of 0.235 for the pair of GigtIV and Sct2-4. Minimum and maximum inter-cluster divergence is 0.314 ± 0.040 (with cluster II) and 0.527 ± 0.061 (with cluster VII), respectively (Table S1). Interestingly, Hct-A and Hct-S actinoporin families do not form separate groups.

Group IA includes only gigantoxin GigtIV from *Stichodactyla gigantea* [73]. It lies at the bottom of the cluster I (bootstrap of 97%) and it has unique substitutions, such as Thr24, Lys131, Asn138, and Lys170 (Figure S2). The inter-group divergence ranges from 0.141 ± 0.038 (with the group IB) to 0.187 ± 0.038 (with the group IC). Group IB is composed of two sequences, Hct-A and Hct-S19, from *H. crispa*. The distinguishing feature is the presence of Gln28, Asp82, and Ser139, as well as Cys88 for Hct-S19. The intra- and inter-group divergence values for IB are 0.048 ± 0.013, and ranges from 0.116 ± 0.026 (with Group IC) to 0.170 ± 0.032 (with Group ID).

The groups IC and ID are the most represented, consisting of 27 and 48 actinoporins, respectively. Group IC only includes *H. crispa* actinoporins in approximately equal numbers of both Hct-S and Hct-A isoforms. The distinguishing feature of IC is the presence of Asn78, Glu82, and Asp96; the majority of sequences have Lys17 and Glu21. This group shows the smallest value of intra-group divergence (0.027 ± 0.011). Group ID is the closest to IC with the divergence of 0.100 ± 0.019. It includes both *H. crispa* and *H. magnifica* actinoporins, which are characterized by the presence of Asp/Glu10, Lys21, and Asp78. The intra-group divergence is 0.071 ± 0.018. Group ID could be divided into four major subgroups, ID1–ID4. Subgroup ID1 is *H. magnifica*-specific, consisting of E6, E13, and G2 with Glu82 and Asp96 like IC actinoporins. Subgroup ID3 includes actinoporins D4, E5, E8, E11, and Hct-S23 with substitutions Gln17 and 172 being found in two other subgroups, ID2 and ID4. Subgroup ID4 is distinguished by the presence of actinoporins with Phe112 in POC site, its role in membrane binding is still being discussed [48,52].

Group IE is composed of sticholysins, StnI and StnII, from *S. helianthus*. It has the largest value of intra-group divergence (0.074 ± 0.022). The level of inter-group divergence ranges from 0.132 ± 0.022 (with the group ID) to 0.182 ± 0.035 (with the group IA). The sequences share substitutions as the cluster I actinoporins have, in addition, they include unique (for the cluster I sequences) amino acids residues: Glu3, Glu17, Val64, Ser78, Ser79, Pro123, and Tyr149 (for StnI), Glu24, Ser96, Glu150 (for StnII), as well as Val18, Thr44, Thr 69, and Trp112.

Despite the differences, all of the obtained actinoporins are characterized by a high content of charged and hydrophobic residues at the N-terminal, what is necessary for α-helix formation and penetration into the lipid membrane [4]. Moreover, the conservativeness of such important for the activity hot spots, as 30SRK32, Lys77, POC-site, and RGD-motive were also found.

Evolutionary analysis of actinoporins revealed the strong influence of purifying selection on majority sites, particularly, being implicated in the structural stability and pore-forming activity [74]. Therefore, the examples of the toxin diversification independent of phylogeny deserve close scrutiny. Our current clustering of sea anemone actinoporins is consistent, in general, with their division into superfamilies and families.

### 2.4. In Silico Analysis

Evidently, actinoporins should exhibit high cytolytic activity in order to fulfill their basic function of lysis of cell membranes. It was demonstrated that their activity is associated with high N-terminal hydrophobicity [11,29,36,42], as well as with the number and distribution of charged residues in this region [20,36,37,40]. It was shown that substitutions of Ala12, Ser13, Lys20, Leu26, Gly27, Asn28, and Arg31 to Cys for EqtII [11,29,36,44], and Lys19Glu for StnII [50], practically did not perform the membrane binding of actinoporins, but significantly reduced pore-forming activity. *H. crispa* actinoporins were found to differ from one another by single substitutions that were localized both in the N-terminal region (1–27 aa) and at the β-core (28–177 aa) (Figure 2). A lot of actinoporin studies are devoted to the investigation of the amphiphilic N-terminal region hydrophobicity [4,20,75]. Here, we focus on the dissection of the molecular surface electrostatic potential and on the revealing of a role of certain charged residues in the pore-formation.

**Figure 4:**
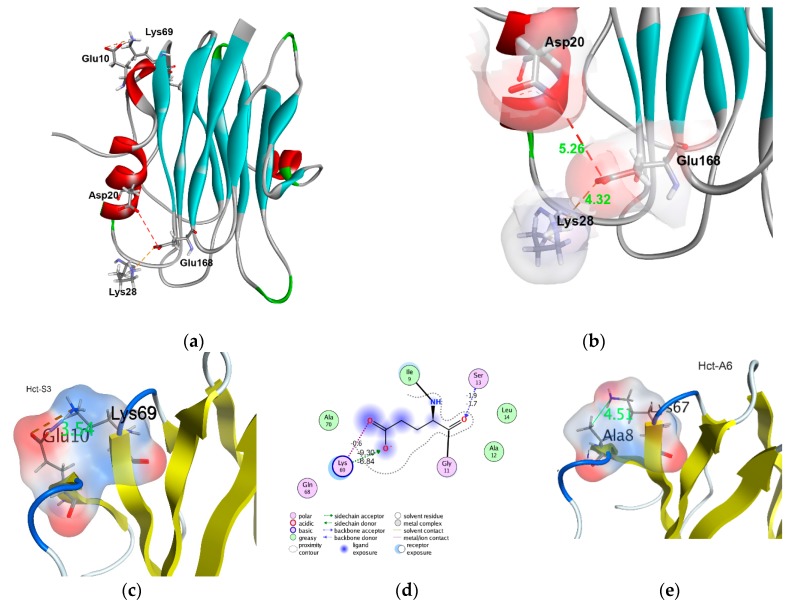

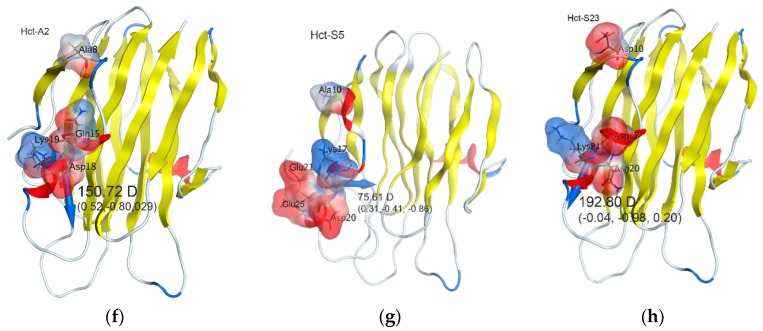
Molecular models of *Heteractis* actinoporins. Ribbon diagrams of 3D-models in water-soluble state: (**a**) Hct-S3, (**b**) Hct-S3 Glu168 residue interactions, (**c**) Hct-S3 Glu10 residue interactions, (**e**) Ala8 and Lys67 interaction of Hct-A6, (**f**) Hct-A2, (**g**) Hct-S5, and (**h**) Hct-S23. (**d**) Two-dimensional (2D) schematic presentation of Hct-S3 Glu10 intramolecular interactions. The dipole moments of the N-terminal fragment (**f**–**h**) are indicated as blue arrow and labeled as value D (Debye) and direction (x, y, z). Variable residues are represented as sticks and labeled, the residue surfaces are colored by electrostatic potential, unfavorable electrostatic interaction is indicated as red dashed line, the salt bridges—as orange dashed lines, hydrogen bonds—as blue dashed lines, the distance both between carboxyl group of Glu10 side chain and Lys69 ε-amino group of Hct-S3 and Ala8 side chain methyl and Lys67 ε-amino group of Hct-A6 are labeled in green. The diagrams were produced using Discovery Studio Visualizer (**a**,**b**) [76] and MOE 2016.0802 (CCG) [77] (**c**–**h**) programs.

Three-dimensional (3D)-structure models were generated and molecular surface electrostatic potentials were calculated for all of the representatives of actinoporins from Cluster I. The atomic coordinates of StnII crystal structure determined with resolution of 1.71 Å [31] and sharing the sequence identity from 90.29 to 99.43% were used as a template. Generated actinoporin models contain twelve antiparallel β-strands and two α-helices that are linked to one another by loops of different lengths (Figure 4a,f–h). The RMSD values for the 175 Cα-atoms of the models did not exceed 0.27 Å relative to the prototype.

According to the results of the pairwise similarity analysis of the molecular electrostatic potential distribution that was performed using the PIPSA method [78], actinoporins form three Clades (Figure S3). This clustering is consistent with the phylogenetic analysis. Therefore, actinoporins from the groups IA, IB, and ID (except Subgroup ID2), as well as Hct-S8 and Hst-S13 from IC and StnII from IE belong to Clade I, actinoporins from the group IC form Clade II, and actinoporins from the subgroup ID2, together with StnI from IE fall to Clade III.

**Figure 5:**
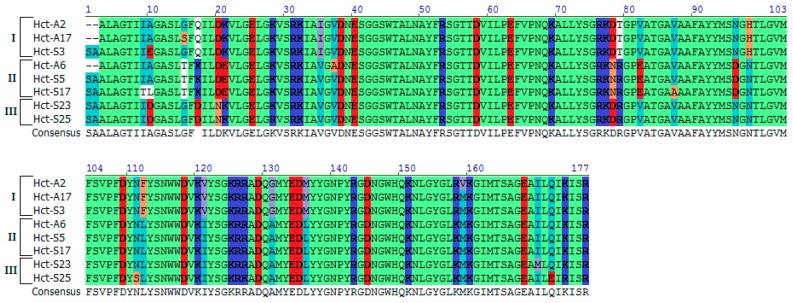
Multiple sequence alignment for *H. crispa* actinoporins belonging to Clades I–III (indicated on the left) according to molecular electrostatic potential distribution. Non-similar amino acids are shown on white background, weakly similar—on light-grown, conservative—on blue, block of similar—on purple, identical—on light-green colors. The positive- and negative-charged amino acids are indicated by navy blue and red colors, respectively.

The analysis of the obtained models revealed several important determinants, which, in our opinion, should contribute to the actinoporin activity; firstly, the residues at the positions 10 and 20, as well as the dipole moment of their N-terminal; secondly, the charged residues (78–82) at the loop connecting β5- and β6-strands. To visualize these results, eight actinoporins from the different Clades were selected. As seen in Figure 5, they have strong differences, especially in these regions.

We revealed Glu/Asp10 forms two salt bridges of ~1.28 ± 0.8 Å and 3.92 ± 0.6 Å lengths, respectively, yielding a total contribution to the binding energy about 7.46 ± 0.15 kcal/mol, with a conserved Lys69 (Figure 4a,c,d). The interaction of these residues is also stabilized by a hydrogen bond with a contribution of −4.8 ± 0.7 kcal/mol. Apparently, it can promote a stronger association of β1-strand to the molecule core that resulted in a more compact packing of the N-terminal fragment when compared to other representatives having Ala/Leu10 (Figure 4e). We assume that this electrostatic interaction may counteract the dissociation of the N-terminal fragment from the β-core after POC-site binding of the actinoporin to the membrane and its immersion into the membrane interface. This agrees well with the increasing hemolytic activity of the mutant StnID9A, and it suggests a key role of the negative charge in providing the strong interaction between the N-terminal fragment and the β-core (namely between Asp9 and Lys68) [79]. Our molecular modeling results suggested that the N-terminal fragment is closely linked to β-sandwich core through an extensive network of electrostatic and hydrophobic interactions, as well as hydrogen bonds. Glu168, conservative for the actinoporins of Cluster I, forms a network of interactions including the ionic one with Lys28. The length and energy of this bond could vary depending on a residue at the position 20. The appearance of Asp at this position leads to unfavorable electrostatic interactions with the similarly charged Glu168. As a result, the density of a positive charge in the membrane recognition region decreases, and the length of the salt bridge between Lys28 and Glu168 elongates from 2.39 ± 0.5 Å for Hct-S23 to 4.32 ± 0.7 Å for Hct-S3 (Figure 4b). This can affect both the rate of the actinoporin association with a membrane and the actinoporin conformation changing during the subsequent pore forming.

This assumption is also supported by a comparative analysis of the electrostatic properties of N-terminal fragment of actinoporin. The results of the molecular modeling, including the full atom MD simulation of the water-soluble form of Hcts, as well as the estimation of their molecular properties that demonstrated that the magnitude, and, more importantly, the direction of dipole moment of the N-terminal fragments of actinoporins from different Clades varied significantly (Table 1). There are some variants of the actinoporin N-terminal fragment dipole moment direction: to the membrane (i), to the molecule β-core (ii), and intermediate positions (iii) (Figure 4f–h). We assume that actinoporins from Clade I, the dipole moments of which are directed to the membrane, should possess higher hemolytic activity than those from the other Clades.

According to cryoelectron microscopy data, residues at the β5–β6 loop that is located on the surface of actinoporin contacts with the lipid interface play an important role, both in the recognition and in the interaction with the membrane. Therefore, the substitutions should definitely affect the binding of the actinoporins to the membrane [31]. Substitutions Asp78Asn and Thr79Arg significantly increase the positive charge density in this area, which contributes to the strong electrostatic interaction of the loop with the membrane surface. In addition, Glu82 (typical for the actinoporins from Clade II) on the one hand can hamper the binding to the membrane and on the other hand stabilize the actinoporin structure in the transition of the molecule from the membrane-bound to the pore-forming state due to its strong electrostatic interaction with the “pseudo-string” 30SRK32 loop. This loop, according to the current concept of actinoporin pore-forming mechanism, plays a key role in the introduction of the N-terminal α-helical region into the membrane by straightening and extending the α-helix [31,37,38,50]. Thus, the substitutions in the β5–β6 loop (Asn78 and Arg79) should promote a better association with the membrane of actinoporins from Clade II than of those from Clade I or III (Figure 5).

### 2.5. Hemolytic Activity of the Recombinant Actinoporins

To test the hypothesis, we compared the hemolytic activity of recombinant actinoporins. The recombinant actinoporins of Clade I (Hct-A3 and Hct-S3) and Clade II (Hct-A5) encoded by high-abundant transcripts were expressed in *E. coli* Rosetta (DE3) strain as His-tagged GST-fusion proteins. According to the electrophoretic analysis, the molecular masses of the fusion proteins were slightly above 50 kDa, which are consistent with the calculated data (~52 kDa). The recombinant actinoporins with molecular masses of approximately 20 kDa were isolated from the cell lysates by affinity chromatography. The N-terminal sequences (15 aa), as determined by sequencing, fully corresponded to those that were deduced from the nucleotide sequences.

The calculated molecular masses, the values of isoelectric points, and the hemolytic activity of the recombinant actinoporins are shown in Table 2. The *H. crispa* actinoporins are high basic polypeptides with close molecular masses. The values of hemolytic activity of actinoporins from Clade I, rHct-А2, rHct-А3, and rHct-S3 corresponded to values of the nature actinoporins, and were on order of magnitude greater than those of actinoporins from Clade II, rHct-A5, rHct-S5, and rHct-S6. It should be noted that rHct-A5 and rHct-S5 exhibit identical hemolytic activity. Their sequences differ from each other only by the presence of additional Ser-Ala at the N-terminus of Hct-S5, which obviously does not affect the activity.

According to the hemolytic activity data and the molecular modeling results, the magnitude and the direction of the N-terminal dipole moment indeed reflects Clade I actinoporins’ ability to possess hemolytic activity, whereas substitutions Asn78 and Arg79 can provide membrane binding of actinoporins from Clade II, while Glu82 plays a key role in their structure stabilizing. We found that the phylogenetic actinoporin clustering corresponded to the values of hemolytic activity of the members. The actinoporins from Subgroup ID4, rHct-A2, rHct-A3, and rHct-S3, have higher activity than rHct-A5, rHct-S5, and rHct-S6 from Group IC (Figure 4). It has recently been found that StnI and StnII can potentiate each other’s activity [80]. This synergy was proved to be caused by the assembly of heteropores. We can assume the diversity of *H. crispa* actinoporins with different pore-forming activity may be important for providing much wider toxicity and specificity in various biotic interactions.

## 3. Materials and Methods

### 3.1. Precursor Determination

cDNA of the sea anemone *H. crispa* was synthesized using mRNA isolated from tentacles of one individual animal by Mint Kit (Evrogen, Moscow, Russia), according to the manufacturers’ recommendations. The rapid amplification of cDNA 3′-ends (3′-RACE) was carried out as described [18]. 5′-RACE was performed with the universal Step-out primer miх1-3 (Mint RACE primer set, Evrogen, Russia) [60] and the reverse primers A1R (TGCACCCGCAATAATT), A2R (AGCGCCCGTTGTATCG), and A3R (ATGCCATCCGTTGTCGCC). The primers were synthesized at Evrogen (Russia).

Amplified PCR fragments were cloned into pTZ57R/T using T/A cloning system (Fermentas, Vilnius, Lithuania). The competent DH5α *E. coli* cells (Stratagene, La Jolla, CA, USA) were transformed by the recombinant plasmids. The white-blue screening was carried out for *E. coli* DH5α recombinant clones selection. The plasmid sequencing was performed on ABI 3130xL Analyzer (Applied Biosystems, Foster City, CA, USA).

### 3.2. Cloning of cDNA Encoding Actinoporins

PCR amplification was performed with hct_sign (5′-TCGTTACc/aATGATA-3′) and hct_notransl (5′-GATTCTCTATTTGTCTTC-3′) primers, Phusion DNA Polymerase (New England Biolabs, Ipswich, MA, USA), and *H. crispa* cDNA as a template under the following conditions: 98 °C for 30 s; followed by 28 cycles of 98 °C for 15 s, 59 °C for 30 s, and 72 °C for 30 s; followed by 72 °C for 15 min. PCR fragments (650 bp) were isolated from agarose gel with a DNA Extraction Kit (Thermo Scientific, Waltham, MA, USA) and cloned into pTZ57R/T using a T/A cloning system (Thermo Scientific, Waltham, MA, USA). *E. coli* strain DH5α cells were transformed by the recombinant plasmids. The presence of a desired insert in ampicillin-selected clones was determined by colony PCR with standard primers. The colonies screening, plasmids isolation, and sequences determination were carried out.

The gene sequences have been deposited in GenBank with the accession IDs MG887781–MG887823 (Figure S1).

### 3.3. Recombinant Actinoporins Production

To generate expression constructs, DNA fragments, encoding Hct-A and Hct-S actinoporins, were amplified with forward hct-af (GGCTTTAGCTGGTACAATTATCGCGGGTGCA) or hct-sf (GTCGGCGGCTTTAGCTGGCACAATTACTCTC) and reverse hct-a/sr (CCCCAAGCTTAGCGTGAGATCTTAATTTGCAGTAT) gene-specific primers, respectively. pTZ57R/T with the *hct-a/s* gene inserts were used as a template. The PCR fragments were digested with *Hind*III and were cloned into the pET-41a(+) vector (Invitrogene, Carlsbad, CA, USA) at the *PshA*I and *Hind*III restriction sites. Recombinant plasmids were used for the transformation of *E. coli* Rosetta (DE3) cells (Invitrogene, Carlsbad, CA, USA) by electroporation on a Multiporator device (Eppendorf, Hamburg, Germany) according to the standard protocol. The transformed cells were cultured overnight in LB medium containing kanamycin (50 μg/mL) and chloramphenicol (34 μg/mL), followed by culturing up to A_600_ = 0.4–0.6. 0.1 mM IPTG (Fermentas, Vilnius, Lithuania) was added for expression induction, and the cells were growing for 20 h at 18 °C. The cells were centrifuged at 6000 rpm and were washed with Tris-HCl buffer, pH 8.0.

Recombinant actinoporins were purified, as described previously [25,26]. The purity of the recombinant actinoporins was confirmed by N-terminal sequencing (10–15 aa) that was performed on Procise 492 cLC protein sequencer (Applied Biosystems, Foster City, CA, USA).

The recombinant proteins were analyzed by Laemmli’s 12.5% SDS-PAGE [81]. Molecular masses were estimated using a PageRuler™ (Fermentas, Vilnius, Lithuania).

### 3.4. Sequences *and Phylogenetic Analysis*

All of the sequences were aligned using ClustalW in MEGA 7 [82] and the alignment is provided in Supplementary materials (Figures S1 and S2, Table S1). Phylogenetic analysis was performed using the Maximum Likelihood method with a gap complete deletion option and bootstrap supporting of 200 replicates in MEGA 7. The best-fit model for protein evolution determined with Modeltest in the MEGA 7 was JTT + G + I. Evolutionary distances of actinoporins were computed using the JTT matrix-based method [73]. Evolutionary distances were calculated according to the JTT matrix-based model with a gamma distribution (shape parameter = 1.7014) on the ungapped multiple alignment positions. The distances refer to the number of amino acid substitutions per site between two protein sequences. The results that are shown are mean values ± standard deviations.

### 3.5. Homology Modeling of Actinoporins

Actinoporins spatial structure models were generated by homology modeling using Modeller 9.11 and Chimera 1.9 programs [76,83]. The spatial structure of StnII (PDB ID 1GWY) from the *S. helianthus* [31], received from the Protein Data Bank, was used as a prototype in constructing the model. The stereochemical quality of the models was tested using PROCHECK [84] Computations of molecular dynamics (MD) simulations for actinoporins in an aqueous environment were performed under conditions of constant pressure, 300 K, and pH 7.0 for 60 ns in an Amber12: EHT force field using the MOE 2016.0802 program (CCG) [77]. The whole system was equilibrated to reduce the initial bad contacts prior to molecular dynamic simulations. Equilibration consisted of energy minimization of the initial side chains’ position with fixed backbone atoms, followed by minimization with restrained carbon alpha atoms and a short molecular dynamic (100 ps). Evaluation of the electrostatic properties of the molecular surface in the Amber Amber12: EHT force field and the visualization of the structures were performed using the MOE 2016.0802 program [77]. The results were obtained using the equipment of Shared Resource Center “Far Eastern Computing Resource” IACP FEB RAS (https://cc.dvo.ru).

### 3.6. Hemolytic Assay

Hemolytic activity was determined on mouse erythrocytes in a medium containing 0.9% NaCl, 1 mM KCl, 10 mM glucose, 5 mM Tris-HCl, pH 7.4, and heparin, as described [16]. The level of hemoglobin in supernatant was measured spectrophotometrically at 540 nm. The amount of the actinoporin causing 50% hemolysis of 0.7% erythrocyte suspension (1 mL) at 37 °C for 30 min was taken as one hemolytic unit (HU_50_).

## 4. Conclusions

We have performed a comprehensive investigation of *H. crispa* actinoporins, including molecular cloning, modeling, and biological activity testing. The approach allowed for us to establish a variety of non-abundant transcripts encoding the sea anemone venom actinoporins. This diversity is determined by the existence of the actinoporin multigene family of 47 representatives at least. It is not still clear if there is a great variation in the number of gene copies among sea anemone species. The transcriptomes of both *H. crispa* and *H. magnifica* appear to contain a large repertoire of similar genes representing the rapid expansion of the actinoporin family due to their duplication with subsequent sequence divergence. The striking similarity between *Heteractis* actinoporins indicates similar mechanisms of generation and isoform diversity maintenance. The major difference between these species is the presence of the most abundant group IC in *H. crispa*. The study of divergence at the isoform level is important for the explanation of their function peculiarities. The phylogenetic analysis and the molecular modeling data demonstrate that the combinatorial library of actinoporins is represented by three groups that are differing from one another by the structural features as well as by the magnitude and direction of the N-terminal dipole moment. The functional analysis of some recombinant actinoporins revealed that *H. crispa* actinoporin grouping was consistent with the different hemolytic activity of their members. We strongly assume that the direction of the N-terminal dipole moment tightly reflects the actinoporins’ ability to possess hemolytic activity.

The synthesis of such a variety of actinoporin isoforms in the same organism may cause an expansion of their specificity to the lipid composition of the target cell membranes (preys and predators). Generally, we believe that the evolutionary advantage of the combinatorial library of actinoporins is the ability to form mixed functional pores for much wider toxicity and specificity in various biotic interactions.

## Figures and Tables

**Table 1:** N-terminal dipole moment of *H. crispa* actinoporins.

Actinoporin	Dipole Moment, Debye	Dipole Moment Direction, x, y, z
Hct-A2	150.72	0.52, −0.80, 0.29
Hct-S3	132.89	0.11, −0.82, 0.56
Hct-A17	102.90	0.45, −0.75, 0.48
Hct-S17	134.30	0.48, −0.72, 0.21
Hct-A6	69.66	0.82, −0.51, −0.07
Hct-S5	75.61	0.31, −0.41, −0.86
Hct-S23	192.80	−0.04, −0.98, 0.20
Hct-S25	159.07	−0.06, −0.91, 0.41

**Table 2:** Physico-chemical characteristics of actinoporins.

Sea anemone	Actinoporin	Molecular Mass, kDa	*pI*	Hemolytic Activity, HU/mg	Reference
*H. crispa*	rHct-S3	19.39	9.31	(3.3 ± 0.31) × 10^4^	
	rHct-A3	19.20	9.75	(2.0 ± 0.49) × 10^4^	
	rHct-A5	19.21	9.45	(1.0 ± 0.26) × 10^3^	
	rHct-A2	19.14	9.76	(4.0 ± 0.36) × 10^4^	[26]
	rHct-S5	19.37	9.33	(1.0 ± 0.53) × 10^3^	[25]
	rHct-S6	19.39	9.10	(4.2 ± 0.52) × 10^3^	[25]
	RTX-S	~20.00	~9.8	5.0 × 10^4^	[16]
	RTX-A	~20.00	~9.8	3.5 × 10^4^	[16]
	RTX-G	~20.00	~10.5	1.0 × 10^4^	[16]
	RTX-S II	19.28	10	3.6 × 10^4^	[17]
*H. magnifica*	HMgI	19.0	9.4	3.6 × 10^4^	[12]
	HMgII	19.0	10.0	3.3 × 10^4^	[12]
*A. equina*	EqtII	19.0	10.5	3.6 × 10^4^	[9]
*S. helianthus*	StnI	19.39	9	2.7 × 10^4^	[15]
	StnII	19.28	9	3.0 × 10^4^	[15]

## Supplementary Materials

The following are available online at http://www.mdpi.com/1660-3397/16/6/183/s1, Figure S1: *Heteractis crispa* actinoporin genes sequences alignment; Figure S2: Actinoporin sequences alignment; Figure S3: Cluster dendrogram of pairwise similarity of the actinoporin molecular electrostatic potential distribution. Table S1: TemporaryMEGA1_JTT pair comparison.
