# Supplementary figures and images for: Multigene Family of Pore-Forming Toxins from Sea Anemone Heteractis crispa
Source: Mar Drugs. 2018 May 24;16(6):183. doi: 10.3390/md16060183 (PMC6025637; doi:10.3390/md16060183)

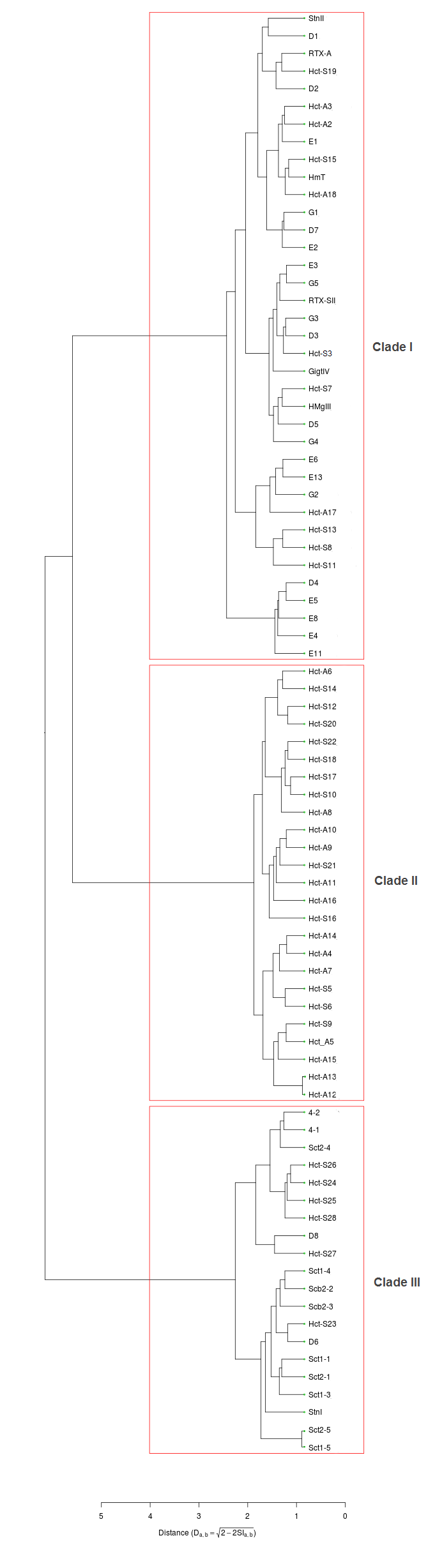

Supplement: Supplementary file 1 [file marinedrugs-16-00183-s001.zip › Supplementary/Figure S3.tif]
